# Advocating for collaboration among key partners to promote diversity in clinical studies amid policy challenges in the United States of America

**DOI:** 10.1186/s13063-025-08820-y

**Published:** 2025-04-02

**Authors:** Samuel Byiringiro, Juliana K. Garcia, Nsenga Farrell, Bunmi Ogungbe, Rifath Ara Alam Barsha, Hailey N. Miller, Evans Whitaker, Paul Wang, William E. Rosa, Barbara E. Bierer, Cheryl R. Himmelfarb, Erin D. Michos, Koen De Lombaert, Maya Berdichesky, Stephan Busque, Latha Palaniappan, Eldrin Lewis, Fatima Rodriguez, Hannah Valantine

**Affiliations:** 1 School of Nursing, Johns Hopkins University, 525 N Wolfe St, Baltimore, MD 21205 USA; 2 Elson S Floyd College of Medicine, Washington State University, Spokane, USA; 3 University of Pennsylvania, Philadelphia, USA; 4 School of Medicine, Johns Hopkins University, Baltimore, USA; 5 Lane Medical Library, Stanford University, Palo Alto, USA; 6 School of Medicine, Stanford University, Stanford, USA; 7 Department of Psychiatry and Behavioral Sciences, Memorial Sloan Kettering Cancer Center, New York, USA; 8 Multi-Regional Clinical Trials Center of Brigham and Women’S Hospital and Harvard, Boston, MA USA; 9 Studypages, San Francisco, USA; 10 Department of Medicine, Brigham and Women’s Hospital and Harvard Medical School, Boston, MA USA

**Keywords:** Clinical research, Clinical trials, Clinical studies, Regulatory institutions, Diversity in clinical research

## Abstract

The lack of diversity in clinical studies has significant ethical and health consequences, limiting the development of effective treatments for diverse populations. Homogeneous participation in clinical studies contributes to health disparities, particularly among historically underrepresented groups in the United States (US). Racial, ethnic, and other minoritized populations have long been excluded from clinical research. In response, the US Congress mandated the National Institutes of Health to assess the impacts of insufficient diversity in clinical studies. Despite efforts by the government, non-profit organizations, and industry players to improve diversity in clinical studies, progress has been slow due to fragmented approaches. For instance, the new US administration (2025) has recently released executive orders which threaten to reverse the progress made in inclusive clinical research. The Stanford Think Tank on Diversity and Equity in Clinical Trials, held in September 2023, brought together key partners across multiple sectors and professions to discuss barriers and explore potential solutions to participation in clinical studies. In this commentary, we discuss the importance of collaborative, inclusive strategies in clinical study design to advance equitable health outcomes for all. Further, we discuss potential implications of the government’s dismissal of diversity, equity, and inclusion initiatives on diverse research participation.

## Background

The lack of diversity in clinical studies is a substantial barrier to continued progress in health science and poses considerable ethical challenges. Clinical studies are key to the discovery of new and continued innovations in existing treatments and interventions and provide the highest evidence base that shape guideline recommendations for disease prevention and management [1, 2]. In the same way a tailored suit might not fit all persons, medical treatments and interventions may not be as effective among all populations. Therefore, clinical study diversification is a critical approach to ensure that medical interventions and treatments work and are safe for all the people for whom they are intended.

Over the past century, major advancements have been made in health sciences. Examples of such innovations include the treatment of previously incurable cancers like leukemia [3, 4], sophisticated treatments of cardiovascular diseases such as coronary heart disease [5], control of deadly viruses like human immunodeficiency virus [6], and improved neonatal and maternal health [7, 8]. However, people with the highest burden of these and other diseases often are not participants in the clinical studies which lead to these discoveries, which can further disparities in health outcomes [9]. For example, despite Black men in the United States (US) having the highest incidence of prostate cancer, they make up only 0.5% of participants in clinical studies for prostate cancer screening [10]. Similar patterns have been observed in clinical studies across various diseases and conditions, including cardiovascular diseases, diabetes, COVID-19, and others [11–13]. Additional populations likely to be excluded from clinical studies include Indigenous and Native American persons and other persons of color; members of certain religious groups; lesbian, gay, bisexual, transgender, and queer (LGBTQ +) persons; persons with disabilities; persons who live in rural areas; and persons otherwise adversely affected by persistent poverty or inequality [9, 14].

Despite significant progress in recent years toward recognizing diversity in clinical research as essential to advancing health sciences, recent (2025) political shifts in the US government have dismantled diversity initiatives and are reversing these gains. However previously, in 2020, the US Congress had mandated the National Institutes of Health (NIH) to sponsor a task force within the National Academy of Sciences, Engineering, and Medicine to assess the long-term health and economic impacts of the lack of diversity in clinical research [9]. Further, over the last decade, the NIH and the Food and Drug Administration (FDA) had released policies for improving diversity in the clinical research that they fund or regulate. Non-profit organizations, professional societies, and for-profit institutions are still investing in promoting diversity in clinical studies. For example, the American Heart Association, a key non-profit organization in the US, has invested $20 million to promote diversity in clinical studies [15]. In January 2025, the new US administration released executive orders abolishing diversity, equity, and inclusion (DEI) initiatives and dismissing health workforce and entire departments charged with DEI across federal offices [16–18]. The FDA diversity action plan guidance slated to take effect in June 2025 was retracted, then restored by the federal judge mandate yet sending mixed message to implementers looking to the government for guidance [19]. Amid these uncertain times, collaboration across key institutions becomes even more important for a unified voice and continued progress in advancing inclusive research.

In September 2023, the Stanford Think Tank on Diversity and Equity in Clinical Trials convened interested parties from academic institutions, industry, the FDA representatives, and the community to engage in thought-provoking discussions on the status of diversity in clinical studies and potential solutions for continuous improvement in diversity in clinical studies [20]. A key topic discussed was the FDA’s role in promoting diversity in clinical studies. Experts and others soon recognized that FDA regulations alone would not be sufficient to advance diversity in clinical studies. Instead, achieving meaningful progress includes collaboration among various entities, including regulatory bodies, clinical research institutions, and community groups. This article explores the recent efforts to enhance diversity in clinical studies, identifies key gaps, explores implications of government’s dismissal of DEI initiatives on diverse research participation, and proposes solutions based on collaboration across key sectors.

## Current state of participation among FDA-regulated clinical studies

### Diversity in FDA-regulated clinical studies

The data on diversity in clinical studies among FDA-approved drugs shows a consistent rise in the representation of women. Among clinical studies leading to FDA approval of drug products between 2014 and 2019, women participation was 51% on average, ranging from 37% in 2014 to 57% in 2019 [9]. An analysis of data from 90 FDA approvals reported in the FDA Drug Trial Snapshots between 2014 and 2021, women have consistently represented over 50% of trial participants in ophthalmology, gastroenterology, endocrinology, metabolism, and bone studies [9]. According to the US Census Bureau, female population was 50.5% of the total US population in 2020—therefore 50% female representation in clinical trials of health conditions that affect men and women equally would be preferable [21]. The most recent Drug Trials Snapshots (2022 and 2023) show similar trends in representation of women in clinical studies at around 50% [22]. However, female representation in oncology trials and trials for therapies to treat heart, blood, kidney, and endocrine disorders that affect both men and women stagnated at 40% [9, 22]. Further, an independent study examining trials that supported FDA approval of cardiometabolic drugs specifically from 2008 to 2017 found women only made up 36% of trial participants [23].

In 2020, Non-Hispanic White (NHW) adults were 58% of the US population, Hispanic people were 20%, Black individuals were 14%, and Asian persons were 6% [21]. The trends of participation based on race showed that among drugs that received FDA approval, the representation of NHW participants trended toward the US population prevalence of that sub-population from 84% in 2014 to 74% in 2020 [9]. Across the clinical trials for 37 drugs approved in 2022 and 55 drugs approved in 2023, the patient cohort predominantly consisted of NHW individuals, with Asian and Black populations following in enrollment numbers [22]. Asian persons comprised ≥ 10% in 27 clinical studies and ≥ 30% in six clinical studies, and Black individuals comprised ≥ 10% of participants in nine clinical studies and ≥ 25% in two clinical studies [22]. Despite notable improvements, however, clinical studies for cardiovascular and endocrine disorders, neurological and psychiatric disorders, cancers, and others show an overall low representation of Black and Hispanic/Latino populations.

### Diversity in NIH-funded clinical trials

Similar to the FDA data, an examination of participation in all types of clinical research and phase 3 clinical trials funded by various NIH institutes and centers indicates a high female representation. The data from the Office of Research on Women’s Health showed that in all NIH-defined phase 3 clinical trials between 2018 and 2022, women participation was roughly 61% [24]. The median representation of Asians, Black Americans, and American Indians/Alaska Natives in phase 3 clinical trials between 2018 and 2022 was 6.1, 20.4, and 0.6%, respectively. At the same time, the median representation of Hispanic/Latino participants in NIH-defined phase 3 clinical trials was 14.6%.

## Efforts and barriers to promote diversity in clinical studies at different institutions

### Clinical study regulatory entities

**Fig. 1:**
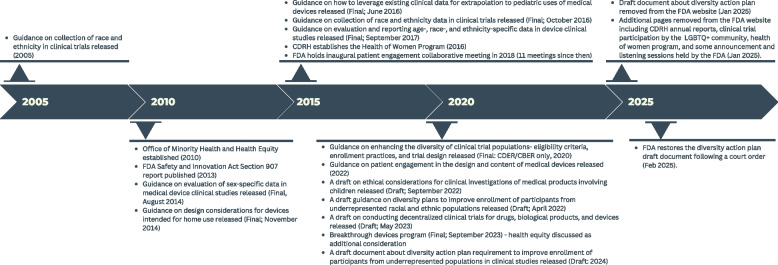
Policy changes and guidance at the Food and Drug Administration to promote diversity in clinical studies

Regulatory agencies in the US, such as the FDA, play a crucial role in safeguarding public health by ensuring that medical products and interventions are safe and effective and clinical studies are conducted ethically. The FDA regulates clinical investigations of medical products, enforces the Federal Food Drug and Cosmetic Act (FD&C Act), and protects users [25]. Given the significant importance of diversity in clinical studies, which, if absent, may limit the strength and generalizability of the evidence for the intended use population, the FDA has introduced several initiatives, some of which are described below (Fig. 1). The initiatives include regularly updated guidance documents for collecting race, sex, gender, and ethnicity data, and the establishment of the Office of Women’s Health in 1994 and the Office of Minority Health and Health Equity in 2010 [26]. These offices focus on research to address health disparities, create educational resources on clinical trial diversity and health equity, and have established the Racial and Ethnic Minority Acceleration Consortium for Health Equity (REACH) in 2023 to efficiently respond to health equity-focused research needs. The Center for Devices and Radiological Health (CDRH) also established health equity as a strategic priority for fiscal years 2022–2025 and engages diverse patients who share their condition and treatment experiences with CDRH staff and the public, through a variety of mechanisms. In April 2022, the FDA issued a draft guidance to provide recommendations on the development of race and ethnicity diversity plans to help enroll historically underrepresented racial and ethnic populations in clinical studies. In June 2024, consistent with the requirements of Sect. 3602 of Food and Drug Omnibus Reform Act of 2022 (FDORA), FDA issued a draft guidance that describes the format and content of Diversity Action Plans, including the timing and process for submitting such plans by application or notification type. Submission of Diversity Action Plans are required under section 505(z) and section 520(g)(9) of the FD&C Act as added by Sect. 3601 of FDORA. Diversity Action Plans are intended to increase enrollment of participants who are members of historically underrepresented populations in clinical studies to help improve the strength and generalizability of the evidence for the intended use population [27, 28].

### Institutional Review Boards

The concept of Institutional Review Boards (IRBs) originated from mid to late twentieth century. Following the National Research Act in 1974, the basic ethical principles were identified for review of research in response to ethical breaches in research, including the US Public Health Service Study of Untreated Syphilis in the Negro Male at Tuskegee and Macon County, Alabama, 1932–1972, and other instances where marginalized populations were exploited in clinical research [29–33]. IRBs are responsible for ensuring compliance with ethical standards in clinical studies by protecting human subjects and ensuring compliance with regulations and institutional policies. In the US, IRBs operate under the oversight of the Office for Human Research Protections (OHRP) within the Department of Health and Human Services (HHS). The work of IRBs is guided by three fundamental ethical principles: respect for persons, beneficence, and justice [34]. These principles help ensure that research participants are treated with dignity, that the risks and benefits of research are carefully balanced and that all groups, particularly those historically underrepresented, have fair access to participate in research.

While IRBs are essential to promote the ethical conduct of research and protect research participants, their protective measures have sometimes led to the unintended consequence of excluding minority and vulnerable populations from research under the guise of protection [35, 36]. Actions to mitigate potential harm to vulnerable populations have unintentionally contributed to a new ethical dilemma—underrepresentation in clinical studies—which has far-reaching implications, as described above. Recognizing this, IRBs have a critical role in shaping research designs to promote diversity by ensuring that study aims, participant selection criteria, recruitment procedures, and other aspects of research are inclusive and just [36–38]. Ensuring that the reviewers are aware and trained on implications for the lack of representativeness in clinical research and that they asses, among other things, accessibility of research by people historically underrepresented is a vital step to addressing the historical exclusion of certain groups and ensuring that the burden and benefits of scientific research are equitably distributed.

### National Institutes of Health and other funders

In the US, clinical studies are sponsored by a range of organizations. A sponsor is responsible for starting and overseeing a clinical trial and often provides the funding. A review of about 135,000 clinical trials over 20 years found that the pharmaceutical industry sponsors the greatest number (36%) of clinical trials, while the NIH and other government agencies sponsor just 3.8% [39]. Around 60% of trials are funded by other sources [39]. As a government agency focused on health equity, the NIH plays an important role in promoting diversity in clinical studies, setting an example for other sponsors.

The NIH’s efforts to increase diversity in clinical studies began with the NIH Revitalization Act of 1993, which established guidelines requiring the inclusion of women and racial and ethnic minorities in clinical research [40, 41]. These guidelines were strengthened in 2001 with an official definition of clinical trials and further updated in 2017 with reporting requirements on clinicaltrials.gov [42, 43]. In addition to policy updates, the NIH has advanced diversity in trials by setting strategic goals in the NIH Minority Health and Health Disparities Strategic Plan 2021–2025 [44], offering targeted funding opportunities and providing educational resources to improve recruitment and retention practices, to name a few examples [45]. Table 1 provides examples of initiatives at NIH to promote diversity in clinical research.

**Table 1:** Selected examples of initiatives and funded projects by the National Institutes of Health designed specifically for promoting diversity in clinical research

Source	Overview	URL
**Reporting and data about diversity in NIH-sponsored or NIH-run clinical research and clinical studies**		
NIH RePORT on Inclusion of Women and Minorities in Clinical Research	Database with diversity information of NIH-funded clinical research and clinical studies since 1994	https://report.nih.gov/research/inclusion-women-and-minorities-clinical-research
NIH Inclusion Data by Research and Disease Category	Reports racial, ethnic, sex/gender representation in in NIH clinical research projects associated with a condition or research category	https://nexus.od.nih.gov/all/2019/05/06/nih-inclusion-data-by-research-and-disease-category-now-available/
**NIH-sponsored research projects with diversity as a core component of their design**		
* All of US* Research Program	A diverse dataset that registered researchers can access to conduct clinical research through analyses of survey data, genomic information, electronic health records, physical measurements, and wearables	https://allofus.nih.gov/about/diversity-and-inclusion
Community Engagement Alliance (CEAL)	Launched to address COVID-19, CEAL aims to address healthcare disparities through trust building	https://covid19community.nih.gov/resources/ensuring-inclusion
Guidance for Participants’ Recruitment and Retention	Summarizes key considerations for recruitment and retention during the planning phase of clinical research	https://www.nimh.nih.gov/funding/grant-writing-and-application-process/points-to-consider-about-recruitment-and-retention-while-preparing-a-clinical-research-study
The National Center for Advancing Translational Sciences Trial Innovation Network	The Trial Innovation Network aims to address critical roadblocks in clinical studies and to accelerate the translation of novel interventions into life-saving therapies	https://ncats.nih.gov/research/research-activities/ctsa/projects/tin https://trialinnovationnetwork.org/elements/who-we-are/
Communities Advancing Research Equity for Health™ (CARE for Health™)	NIH is establishing a network to advance research in primary care settings	https://commonfund.nih.gov/clinical-research-primary-care

### Academic institutions conducting clinical studies

Academic institutions play a critical role in advancing scientific discovery. Thus, commitment and action from academic researchers are essential to improve diversity in clinical studies they conduct and promoting the science diversity in clinical research. Evidence from various academic research shows that a combination of multiple strategies might be required to promote diverse recruitment into clinical studies. Some of the recommended strategies include training researchers on cultural competency, community partnerships, personalized approach to recruitment and follow-up, and tackling logistical barriers (transportation, scheduling, et al.) [46]. Researchers at Stanford University demonstrated that letters with language tailored to specific ethnic groups were more effective in increasing minority enrollment than generic letters [47]. Other work in this area is ongoing. For example, one of the projects sponsored by the American Heart Association is evaluating a community-informed text messaging intervention to educate people on clinical studies and share ongoing research opportunities [48].

Diversity among trial investigators and study team conducting the research has shown a correlation in successful recruitment of people represented in the research team [49, 50]. Academic institutions are positioned to promote diversity in clinical studies by training individuals from historically underrepresented populations. Training Researchers to Advance Inclusion Networks (TRAIN) Center, a collaborative between Stanford University School of Medicine and Morehouse School of Medicine offers a structured curriculum to post-doctoral fellows on promoting diversity in clinical studies [51]. Other centers offering career growth opportunities for researchers from underrepresented backgrounds include Creating Opportunities for Underrepresented Researchers to Achieve Growth and Excellence (COURAGE) and Supporting, Promoting and Launching the Expansion of Nutrition, Diabetes, and Obesity Researchers in North Carolina (SPLENDOR-NC) [52].

### Pharmaceutical industry

The pharmaceutical industry is a key sponsor and implementer of drug trials. Drug trials supported by the pharmaceutical industry are driven by the imperative to develop and commercialize new therapies. The overarching aim is to secure regulatory approval from bodies such as the FDA or the European Medical Agency (EMA), which paves the way for market entry. Pharmaceutical trials are characterized by substantial financial investments and a methodical approach to managing research. The industry often collaborates with contract research organizations (CROs) to ensure compliance with regulatory standards and to streamline trial operations. These clinical trials typically involve large-scale, multi-center studies designed to enhance generalizability and meet market demands. Despite their strengths, pharmaceutical trials increasingly face scrutiny over potential biases, including selective reporting and the exclusion of certain patient demographics, [53] which can affect the real-world applicability of their findings.

Many pharmaceutical companies have started working to improve clinical trial diversity within their studies. In fact, a January 2021 survey among 31 pharmaceutical companies revealed that 97% reported taking specific measures to address access issues for clinical trial participants [54]. Additionally, 87% of these companies were adapting protocol design to increase diversity, including the use of decentralized trials, remote trials, and mobile technology, while 84% reported efforts to increase patient education and awareness of clinical trials [54]. The five key strategies being implemented by pharmaceutical industry to enhance diversity in clinical trials are outlined in a 2022 PhRMA report [55]. These strategies are (1) to create networks of clinical trial sites in underserved communities, (2) to put a focus on developing a diverse pool of investigators and staff who reflect the communities they serve, (3) to establish long-term relationships through community-building efforts (health education and support for future diverse health practitioners), (4) to engage communities in open conversations about the importance of trial participation and maintaining transparency throughout the process, and (5) to use of standardized platforms, including the use of real-world data and improved race and ethnicity data [13].

### Community and community advocacy groups

There is consensus from academic institutions, the pharmaceutical industry, key funders, and regulatory institutions that investment in our communities and community-engaged research is essential to achieve diversity in clinical studies. Despite various historical events that have led to mistrust in clinical research, many community members are still willing to participate in research if it is conducted in an ethical and transparent manner. All efforts should be aimed at aspiring to become community allies, promote inclusivity, and re-build trust [56].

Community-engaged research exists on a continuum from no community involvement to community-driven research [57]. Community-based participatory research is one point on the continuum in which researchers and community members are full partners from ideation of the research idea to the dissemination of findings [57]. Depending on the existing level of trust between researchers and the community, lower or higher levels of engagement can be deployed. Higher levels of community engagement allow higher access and recruitment of research participants from the engaged communities [58]. Some of the strategies for acquiring community feedback or partnering with them on research projects are listed below.

- *Community advisory boards* (CABs) have emerged as an effective means of engaging communities through researcher-CAB partnerships in clinical research and promoting diversity. They serve as liaisons between researchers and the community, providing input as full partners on research priorities, question development, study design, recruitment strategies, dissemination of results, and long-term community benefit [59, 60]. By involving community members in the research process, CABs or advisors help build trust, increase transparency, and ensure that studies are responsive to community needs and priorities.
- *Patient advocacy organizations* focused on specific diseases or conditions can be valuable partners in promoting diversity in clinical studies. Some of these organizations have established relationships with patients and caregivers and these can help identify and address barriers to participation. For example, the Alzheimer’s Association has implemented initiatives to increase the enrollment of underrepresented groups in Alzheimer’s clinical studies, such as providing education and outreach to minority communities and partnering with researchers to develop culturally appropriate recruitment materials [61].
- *Faith-based organizations* play a key role in engaging underrepresented communities in clinical research. These organizations are often trusted sources of information and support within their communities. Partnering with faith leaders to educate congregants about clinical studies and encourage participation can be an effective strategy for increasing diversity [62]. For instance, several studies successfully enrolled Black participants in clinical studies by engaging and recruiting in African American dominated churches [63–65].

Additional strategies that have shown to be effective in acquiring community feedback include engaging patient navigators or community health workers (CHWs) [66] and academic-community partnerships [67, 68].

## The role of technology in promoting diverse participation

Technology plays a crucial role in promoting diversity in clinical studies by enhancing the accessibility, recruitment, and engagement of underrepresented populations. Key strategies applied in clinical studies include multimedia (video, slides, websites) presentations of study materials, mobile applications such as the use of clinical trial management software, social media for targeted recruitment, machine learning and Electronic Health Record data mining to identify eligible participants, and electronic consent, virtual meetings to name a few [69]. These technologies can facilitate more inclusive recruitment by helping researchers engage easily with diverse participants.

Prior studies have focused on the actual and potential dangers of technology such as the loss of privacy, misinformation, erroneous data, and so on, in study recruitment and ongoing participant engagement, yet, seldom on solutions for making technology an integral tool for diversifying clinical studies [70]. The researchers across sectors and industries have an opportunity to work together to devise strategies for making technology a tool for increasing diverse clinical study enrollment. Crucial to long-term adoption and success of technology recruitment and ongoing participant engagement strategies is an assessment of digital knowledge and capabilities, access to the internet and necessary digital technology, digital divide, and user-friendliness of digital tools. Without proper assessment and careful enrollment, technologies can invoke distrust and disparately low enrollment among populations with a lack of trust in or access to the technology utilized in the clinical studies [71].

## Potential implications of reversing diversity initiatives on clinical research

In January and February 2025, the federal government released multiple executive orders many of which targeted diversity initiatives [16–18, 72]. Across all federal government offices, the executive orders mandated the firing of employees and dismantling departments focused on DEI programs—deeming them wasteful [73]. At the FDA, the Diversity Action Plan guidance, which was still in draft form and scheduled to take effect in June 2025, was suddenly retracted from the FDA website without notice [19]. Despite restoration of the guidance under court orders, messaging annexed with it notifying viewers that the federal government rejects the guidance and its content is discouraging for implementers looking for government guidance to improve diversity in clinical research. Multiple other pages were taken down from the FDA website including the health of women program, CDRH 2024 report, increasing clinical trial participation for the LGBTQ + community, and Oncology Center of Excellence Equity program [19]. Within the NIH, the executive orders to dismantle DEI initiatives included, among other things, ending NIH equity-related grants, diversity supplements to train scientists from underrepresented minority groups, and the abolition of certain terminologies that have to do with DEI [74].

These changes have deep cross-sectoral implications and risk reversing the progress which had been made in advancing diversity in clinical research. The requirements of the diversity action plan cannot be successful without the government to evaluate the initial plans, monitor their implementation, offer guidance, and enforce the law. The lack of NIH funding for diversity initiatives will lead to failure to train researchers from historically underrepresented groups by academic institutions and inability to build community-academic partnerships which are crucial for increasing research awareness and build trust in research, to name a few. The demonization of diversity terminologies may propagate from government to the private sector thus reversing any work to invest in building infrastructure for diverse clinical research.

Diversity in clinical research is integral to the rigor of the science itself. Non-diverse clinical research risks undermining external validity—rendering findings ungeneralizable and applicable only to a select group of participants who were represented. Further, this robs scientists of the opportunity to understand the effect of different interventions among different population groups, including potentially groundbreaking understandings of the nature of human body and behavior while assessing safety.

## Additional collaborative solutions

**Fig. 2:**
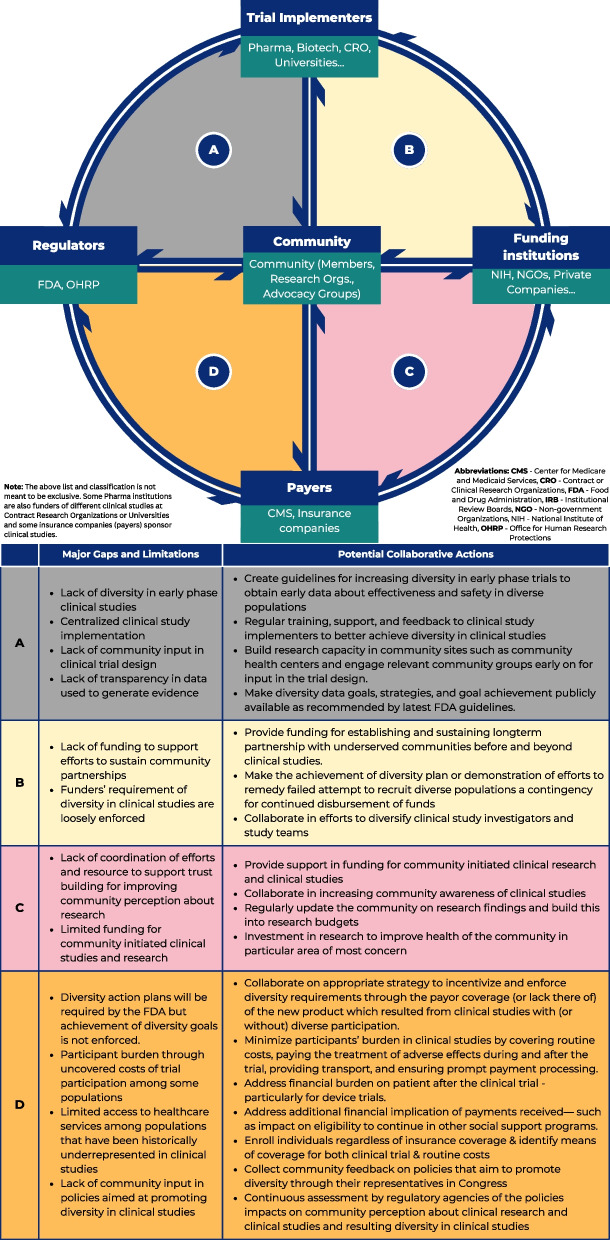
Potential collaborative solutions to the lack of diversity in clinical studies in the US

Despite promising strategies for promoting diversity in clinical studies by different entities, gaps remain in many foundational areas among regulators, funders, clinical trial implementers, and the community. One significant gap is the suboptimal quality of or complete lack of collaboration among key partners, resulting in unsustainable gains and further breach in community trust in academic institutions [75, 76]. More rigorous evaluation and dissemination of effective community engagement strategies would also be helpful. Many successful initiatives remain localized and are not widely adopted due to a lack of evidence or awareness [77]. Collaborations aimed at community empowerment, cultural learning, workforce diversity, transparency, and scientific excellence can sustainably promote diversity in clinical studies. Figure 2 provides a list of potential collaborative solutions to some of the existing gaps that lead to the lack of diversity in clinical studies in the US.

In the collaboration aspect, the FDA had previously requested public comment from interested parties on the draft guidance “Diversity Action Plans to Improve Enrollment of Participants from Underrepresented Populations in Clinical Studies”, prior to finalization of the guidance. The guidance will assist sponsors conducting certain clinical studies to meet requirements for submission of Diversity Action Plans under section 505(z) and section 520(g)(9) of the FD&C Act as added by Sect. 3601 of FDORA. If the feedback from key collaborators such as clinical trial sponsors and the community is considered and integrated accordingly, the guidance may have increased pragmatism, feasibility, and widespread adoption across research settings. This effort could be more effective with regular training provided for clinical trial implementers on scientifically proven strategies for promoting diversity. However, the draft guidance providing recommendations for the form and content of the submission of diversity action plans has faced criticism for lacking sufficient incentives (tax credit, fast regulatory review, and others) for implementers of clinical studies to promote diversity. In addition, there have been calls of developing additional regulations for clinical studies that fail to meet diversity recruitment goals [78, 79]. Further collaborative efforts could help address this issue. For example, making diverse participation a significant factor in the approval process for medical products or linking it to insurance coverage decisions could be a potential partnership between the FDA and payor institutions, encouraging greater investment in diversity in clinical studies by sponsors.

Despite our focus on diversity in clinical studies in US populations, countries outside those in North America and Europe have limited access to clinical studies, which limits their contribution to the advancement of medical science—even though at different levels, they use products of those clinical studies [13]. The importance of lacking representation in clinical studies is of significance to international policymakers and national or international manufacturers, government and non-government organizations. Global collaborations among leading pharmaceutical companies, academic institutions, country representatives, non-profit organizations, and other key stakeholders could be formed to promote local infrastructures for global access to clinical studies and their findings. These efforts would be beneficial to all stakeholders involved as they open new markets for the pharmaceutical industry, promote clinical trial enrollment goals by accessing diverse populations around the globe, and help local communities get access to the latest medical discoveries.

## Conclusion

Engaging community members and advocacy groups is essential for increasing diversity in clinical studies. By leveraging the expertise and trust of these groups, researchers can build relationships, address barriers, and create a more inclusive and equitable clinical research enterprise. Congress can support these efforts by passing legislation that promote diversity, reduce financial burdens, and increase public awareness of clinical studies. However, gaps remain in diversifying the research workforce, providing adequate resources for community engagement, and disseminating effective strategies. These gaps are further amplified by the mixed messaging from the changing government administrations. Addressing these challenges requires a concerted effort from all key partners, including researchers, funders (government, private, and industry funders alike), policymakers, and most importantly, the community. Only by working together can we advance equitable health outcomes for all.

## Data Availability

Not applicable.
